# Electrospun-Fibrous-Architecture-Mediated Non-Viral Gene Therapy Drug Delivery in Regenerative Medicine

**DOI:** 10.3390/polym14132647

**Published:** 2022-06-29

**Authors:** Elena Cojocaru, Jana Ghitman, Raluca Stan

**Affiliations:** 1 Advanced Polymer Materials Group, University Politehnica of Bucharest, 1-7 Gh Polizu Street, 011061 Bucharest, Romania; elena.cojocaru3105@upb.ro; 2 Department of Organic Chemistry “C. Nenitzescu”, University Politehnica of Bucharest, 1-7 Gh Polizu Street, 011061 Bucharest, Romania; rl_stan2000@yahoo.com

**Keywords:** electrospun fibrous architecture, gene therapy drugs, sustained release, transfection efficiency, therapeutic outcomes

## Abstract

Gene-based therapy represents the latest advancement in medical biotechnology. The principle behind this innovative approach is to introduce genetic material into specific cells and tissues to stimulate or inhibit key signaling pathways. Although enormous progress has been achieved in the field of gene-based therapy, challenges connected to some physiological impediments (e.g., low stability or the inability to pass the cell membrane and to transport to the desired intracellular compartments) still obstruct the exploitation of its full potential in clinical practices. The integration of gene delivery technologies with electrospun fibrous architectures represents a potent strategy that may tackle the problems of stability and local gene delivery, being capable to promote a controlled and proficient release and expression of therapeutic genes in the targeted cells, improving the therapeutic outcomes. This review aims to outline the impact of electrospun-fibrous-architecture-mediated gene therapy drug delivery, and it emphatically discusses the latest advancements in their formulation and the therapeutic outcomes of these systems in different fields of regenerative medicine, along with the main challenges faced towards the translation of promising academic results into tangible products with clinical application.

## 1. Introduction

Gene-based therapy is considered one of the most revolutionary technology approaches for various biomedical applications that has advanced along with DNA recombination technology and gene cloning technology [1]. Unlike targeted or conventional drug therapy, gene-based therapy acts at the DNA or mRNA level to intentionally modulate gene expression in specific cells for preventive or therapeutic actions through correcting gene transcription and translation processes [1], affording long-lasting and curative benefits in the treatment of various inherited and acquired diseases [2,3,4]. Considering that gene-based therapy implies the introduction of a functional tunable therapeutic gene to directly repair/amend or replace the altered genetic material at the molecular level [5,6], this unique approach may facilitate the modulation of genetic information through the exogenous stimulation of key signaling pathways in targeted cells, attaining various intended functions (e.g., the differentiation of certain cells into specialized cells, the production of cellular therapeutics or the stimulation of the apoptosis process in cancer cells) and offering innovative approaches for improving the targeted functions as well as fostering both the advancement of new therapeutics based on cell gene correction and their clinical translation [4,7].

Currently, the “library” of gene therapy drugs mainly comprises plasmid DNA (pDNA), small interfering RNA (siRNA), microRNA (miRNA) and short hairpin RNA (shRNA) along with antisense oligonucleotides (ASO) [1,8]. Nonetheless, exploiting the full potential of gene-based therapy in academic or clinical practices entails certain strategies that are able to tackle the main drawbacks faced in gene delivery (e.g., low stability and serum protein interactions, recognition by immunologically active factors, reduced targeting and cell uptake abilities, low endosomal escape and reduced transfection activity) [1,2,9]. Therefore, the selection of an appropriate gene delivery carrier that is capable of specifically targeting selected cells, avoiding the stimulation of the innate immune system or toxicities and proficiently passing through complex intracellular barriers to safely reach the nucleus, protecting the incorporated gene from extracellular or intracellular enzymes and consequently advancing therapeutic efficacy represents one of the most critical aspects in gene therapy drug delivery [10,11]. To date, a plethora of carrier-based approaches, comprising bio-inspired biochemical assemblies of molecular to nanoscale dimensions, generally categorized into viral and non-viral vectors (e.g., reconstructed viruses [9,12], vesicles or nanoparticles [6]) have been engineered and investigated as potent carriers for the efficient and safe intracellular delivery of gene therapy drugs without hampering their therapeutic performances [8,13,14]. However, the direct administration of gene therapy drug carriers may determine systemic distribution in the body, generating risks of gene expression in non-targeted/normal cells, whereas the repeated and periodic administration of carriers, which can represent the main route to ensure and extend gene expression and therapeutic outcomes, respectively, may be inconvenient for patients [2,15].

In this respect, the combination of gene delivery technologies with electrospun fibrous architectures represents an amenable and potent strategy that may address the aforementioned concerns, considering that electrospun fibrous architectures as rationally designed gene therapy drug delivery templates are capable of ensuring not only physical support and guidance for cells but also the controlled and local release of gene therapy drugs in accordance with the therapeutic purpose, its pharmacological properties and patient-specific needs [16,17]. 

Currently, owing to the high tunability of physical properties and versatility in formulation, electrospun fibrous architectures are valuable materials with a diverse array of applications, serving as sensors [18] and catalysts for H_2_ production [19], environmental protection devices [20,21] or energy conservation and storage platforms [22]. The multifarious design, good biocompatibility, reduced immune response and ability to incorporate different types of bioactive molecules [23] make them versatile biomaterial platforms, which are of particular interest to tissue engineering and regenerative medicine, being investigated as potent biomaterials for repair and the regeneration of skin, bone, nerves, etc. [24,25,26,27,28]. Furthermore, the nanofibrous architectures of these systems can efficiently mimic the structure and functions of the native extracellular matrix (ECM), working as highly effective interfaces to retain the cellular morphologies as well as to deliver the incorporated bioactives at the necessary rate to the target site/cells [29,30]. As a spatial template for gene delivery, electrospun-fibrous-architecture-mediated gene delivery is able to modulate the spatial and temporal release kinetics of gene vectors, improving the therapeutic outcomes of gene therapy [2,15,31]. Moreover, beside the high spatial and temporal control of the incorporated gene originating from their ultrathin fiber diameter and large surface-volume ratio, electrospun fibrous architectures are capable of stimulating the seeded cells to proliferate and differentiate during an ex vivo pre-culture period, therefore promoting tissue formation after implantation in vivo [10,32]. In addition, the electrospun fibrous architectures will continue to release incorporated genes after implantation, improving the desired physiological response, respectively influencing surrounding tissue regeneration in the defect area, primordial features in regenerative medicine. 

This review primarily describes the gene therapy drug delivery system, including viral and non-viral vectors along with their strengths and opportunities, particularly focusing on non-viral vectors as compelling gene therapy drug carriers and narrating the most encountered non-viral vehicles. Then, the powerfulness of the electrospinning method in the generation of fibrous architectures, the most important strategies in the combination of electrospun fibrous architectures with gene therapy drug delivery and the main advantages and downsides of each approach along with the in vitro and in vivo therapeutic performances of electrospun-fibrous-architecture-mediated gene therapy drug delivery are summarized. Finally, the current application progress of electrospun-fibrous-architecture-mediated gene therapy drug delivery in tissue engineering and regenerative medicine along with the main opportunities and challenges faced toward the translation of promising academic results into tangible products with clinical applications are also discussed from multiple perspectives.

## 2. Non-Viral Vector Gene Therapy Drug Delivery Platform

The fundamental engineering challenge of gene-based therapy is the construction of a safe and efficient delivery vector. Both viral and non-viral vectors have been extensively used for systemic delivery in academic research and clinical trials [14]. In fact, a large part of gene therapy clinical trials performed so far are based on modified virus-based vectors (retroviruses, lentiviruses, adenoviruses and adeno-associated viruses), which are considered to be the heart of this dichotomous field, allowing researchers and clinicians to develop powerful drug platforms and thus radically changing the face of medicine [33]. Although this type of gene therapy drug delivery has substantially advanced the field of gene therapy, some major limitations are associated with viral-based vectors, including carcinogenesis, immunogenicity, broad tropism, limited gene-packaging capacity and difficulty of mass vector production [12,14,33]. As an alternative to controversial viruses, non-viral gene therapy drug delivery has the potential to tackle many of the limitations of viral vectors, particularly with respect to safety, the ability to deliver larger genetic payloads and the simplicity of synthesis, as compared to viral counterparts [34,35]. One of the most important particularities of non-viral vectors is the protection of carried gene therapy drugs against premature degradation in physiological fluids and extracellular space before reaching the site of action, which represents the driving factor in improving circulation time, intracellular transfection efficiency and therapeutic outcomes, respectively [36]. The main strengths, challenges and opportunities of viral and non-viral vectors in gene therapy drug delivery are presented in Table 1. 

**Figure 1:**
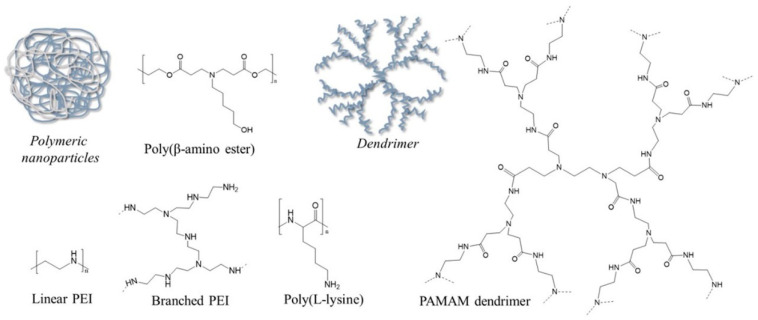
Chemical structure of the most commonly used polyplexes as non-viral vectors in gene therapy drug delivery studies and clinical trials. PEI and PLL are among the oldest and the most used non-viral vectors.

Motivated by the limitations of the viral counterparts, a diverse collection of synthetic non-viral vectors has been developed to bring therapeutic genes to the sites of action, including cationic lipids and polymers (e.g., cholesterol, DC-cholesterol, polyethyleneimine (PEI), PMMA dendrimers, chitosan or cationic cellulose) [11,14,40], peptides (e.g., lysine or arginine) [6,41] and inorganic nanoparticles (e.g., gold nanoparticle, metal-organic frameworks) [42]. Although non-viral vectors are good candidates for gene therapy drug delivery, their relatively limited transfection efficiency as compared to viruses, reduced specificity and, in some cases, low biodegradability still limit their clinical translation [37]. Since viruses have advanced to deliver their genomes efficiently to mammalian cells, most synthetic vectors are unable to effectively transport their payloads over the multiple barriers encountered during delivery [14,43]. Hence, the fundamental factors that guide successful gene therapy drug delivery mediated by non-viral vector strategies are the formulation and stability of carriers, their affinity to the cellular membrane, the internalization of carriers through one of the endocytic pathways as well as their endosomal escape along with their efficient disassembly and gene decomplexation, in some cases [43,44]. Among the variety of non-viral vectors described in the literature, polyplexes—interpolyelectrolyte complexes—spontaneously formed through the electrostatic condensation of nucleic acids and cationic polymers have attracted great attention from the biomedical field [44,45], owing to better safe profiles and easier interactions with negatively charged oligonucleotides and cellular membranes, as well as immense chemical diversity and high potential for functionalization [46]. The chemical structures of the most encountered polyplex-mediated gene therapy drug delivery in tissue engineering applications are presented in Figure 1. 

Polyethylene imine (PEI) and poly-l-lysine (PLL) (Figure 1) are among the oldest and most popular synthetic polymeric vectors for gene therapy drug delivery with applications in different biomedical fields. PLL is the homopolypeptide of the basic amino acid lysine with good stability, safety and water-solubility in weak-acidic or neutral conditions. However, it has been recognized that the PLL-based vectors are characterized by a relatively low transfection efficacy because of their inefficient release from endosomes, as well as their rapid removal from systemic circulation originated from their high susceptibility toward plasma proteins [44].

Otherwise, PEI is a positively charged synthetic polymer widely used in the delivery of different genetic materials, which, beside the protection following administration, is also capable of selectively delivering and releasing the loaded bioactives inside the cytoplasm, improving the therapeutic activity of the system [47]. Containing a particular arrangement of amino groups along the backbone chain, PEI has an extraordinary cationic charge density (at reduced pH values) and buffering capacity, which are postulated to be underlying factors in its complexation abilities with nucleic acids (DNA/RNA) and endosomal/lysosomal release via the “proton-sponge” effect [48,49]. The formulation of PEI-based vectors for gene therapy drug delivery, along with their ability to promote gene transfection in vitro and in vivo, was pioneered by Boussif and co-workers in 1995 [50]. Soon after, a wide variety of PEI-based carriers were formulated and extensively investigated as non-viral vectors for gene therapy drug delivery, showing that the transfection activity and cytotoxic profiles of PEI are highly dependent on its structural features, e.g., molecular weight [51] and conformation (linear versus branched form) [52] (Figure 1). 

It is well-known that the surface physico-chemical properties of synthetic materials (e.g., hydrophilic-hydrophobic properties, chemical composition and charge density) are fundamental factors in the regulation process of interfacial interactions, its biological behavior and cellular responses [51]. Therefore, the intrinsic cytotoxicity of unmodified PEI originating from the strong cationic charge density and degree of branching represents a serious concern in biomaterial application and clinical use [16,44,51]. In this respect, numerous approaches have been deployed, showing that chemically modified PEI derivatives may overcome some of these shortcomings, being capable of improving in vivo efficacy and tolerability as well as transfection efficiency and targeting ability to specific cells or populations [13,53]. For instance, polyethylene glycol (PEG)-grafted PEI formulation condensing mRNA shows remarkable transgene expression levels in pulmonary immune cells following systemic administration [54], and chemically modified PEI with chondrocyte-affinity peptides [55] or cyclodextrin-PEI conjugates [56] have been demonstrated to be potent and feasible gene carriers in vivo. Hence, PEI still continues to be considered the “gold standard” in non-viral gene therapy drug delivery, owing to its good transfection efficiency, which may be further improved through functionalization with a wide diversity of biomolecules/compounds [57,58], and the high interest of PEI as a non-viral vector in the transient gene expression of mammalian cells in vitro and in vivo is reflected in numerous research studies reported in the literature, some of them being under different phases of clinical investigation (Table 2).

However, to address the concerns of efficiency and toxicity associated with PEI and PLL, other polymers have been preclinically investigated as gene therapy drug delivery carriers, including poly(β-amino ester)s (PBAE) and various dendrimers (Figure 1). The PBAE “libraries” contain cationic amines and biodegradable ester bonds in the main structure and have been designed to present improved biodegradation and reduced cytotoxicity [53]. PBAE-based vectors have been shown to have good potential in delivering DNA in pulmonary cells [59] or siRNA in human orthotopic glioblastoma [60], and very recently, Blanchard et al. [61] have proven that PBAE-based carriers are capable of effectively delivering mRNA-encoded Cas13a in rodents for mitigating influenza and SARS-CoV-2 infections. Polyamidoamine (PAMAM) dendrimers, which are nano-sized polymers with versatile structures, a high degree of intermolecular uniformity and multiple terminal functionalities, are another class of polycationic polymers employed in polyplexes construction, since they present lower immunogenicity and toxicity and high transfection efficiency as compared to other cation polymers (Figure 1) [44,53]. The main shortcoming of these types of non-viral vectors is the rapid clearance from systemic circulation that may be overcome through functionalization with short oligopeptides [62]. Dendrimer-based polyplexes have proven to be good strategies in gene therapy drug delivery, successfully mediating siRNA delivery to hepatic cells [63] or drug and pDNA/siRNA co-delivery to different cancerous cells [64].

Although tremendous progress has been achieved in the field of gene therapy drug delivery, challenges related to rapid clearance after introduction in biological environments and insufficient understanding of biodistribution and pharmacokinetics following systemic administration still impede the clinical translation of many potential non-viral delivery systems. To address these concerns, novel technologies that can modulate delivery routes and profiles, in accordance with targeted applications, and that can facilitate the successful translation to clinical use, have been explored. The combination of non-viral gene delivery technology with polymeric scaffolds represents a potent strategy that enables the localized and controlled release and expression of therapeutic genes, enhancing gene transfection efficiency and therapeutic outcomes in comparison to conventional delivery methods (e.g., direct administration) [5]. To date, a wide variety of scaffolds with different architectural configurations and morphologies have been constructed and investigated for gene therapy drug delivery, and, among them, scaffolds with nanofibrous architectures present a particular interest to biomedicine that may increase the potency of gene therapy in both academia and pharmaceutical/clinical practice [2,15,31,65].

## 3. Engineering the Electrospun Fibrous Architecture for Gene Therapy Drug Delivery

### 3.1. Relevance of Electrospinning Techniques in Regenerative Medicine

Electrospinning represents an innovative and unique but also straightforward and versatile technology that uses high electrostatic forces to generate ultrathin polymer fibrous structures with nano- or submicron dimensions that morphologically resemble the natural extracellular matrix (ECM) (the crucial extracellular component that surrounds and acts as a physical environment for cells) [32] and specific features for biomedical applications [2,66,67]. Since the architectural and morphological characteristics of electrospun structures can be greatly modulated and controlled during the electrospinning process by adapting certain parameters (e.g., the applied electric voltage, the solution flow rate, collecting the distance between the needle tip and the target, the geometry and rotation speed of the collector or the needle movement speed), solution properties (e.g., polymeric solution viscosity, polymer concentration, solution surface tension, surface charge, electrical conductivity and the dielectric constant) or environmental parameters (e.g., temperature, humidity) [30,68], this technique has attracted worldwide research interest, being used in different fields of biomedicine and biotechnology. Owing to their multifarious morphological designs (e.g., hollow, core-shell, porous, beaded and ribbon-like structures), versatility in the incorporation of diverse bioactives (e.g., drug molecules, genes or proteins) [69], large specific surface area, high interconnected porosity and permeability along with their ability to efficiently deliver the loaded cargoes in a high spatial and temporal regulated manner, these structures are ubiquitous in tissue engineering [26,70,71], controlled drug delivery [72,73,74] and gene therapy [75,76,77]. 

Acting as a reservoir, beside the ability to ensure a gradual release kinetics of incorporated non-viral vectors, the electrospun fibrous architectures protect vectors carrying genes from premature clearance, protect against degradation in biological environments and protect against extracellular barriers [13], acting also as a platform for regeneration, providing temporary support for cell development in the surrounding environment. Supplementary physical three-dimensional support ensured for cell constituents, the intrinsic mechanical and biochemical elements of matrices may modulate cell phenotypes and functions and are essential in tissue morphogenesis, differentiation, homeostasis and response to injury [78]. By manipulating the design or the physico-chemical properties of fibrous matrices or by modifying the molecular interactions between gene vectors and polymer fibers through their surface properties, the release kinetics of the incorporated gene vectors may be modulated and controlled, ranging from a few hours to several months, thus prolonging the existence time within the cellular microenvironment, improving the gene transfer efficiency and extending the period of gene expression in accordance with the therapeutic purpose [79,80].

### 3.2. Fabrication Methods

**Figure 2:**
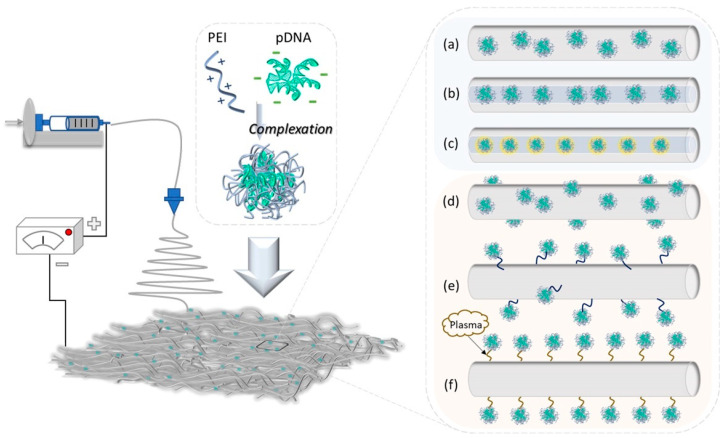
Fabrication strategies of electrospun fibrous architectures for controlled gene therapy drug delivery. The genetic material can be loaded within the electrospun fibrous architectures by encapsulation using: (**a**) blend electrospinning; (**b**) coaxial electrospinning; and (**c**) emulsion electrospinning, or it may be immobilized on the surface of preformulated electrospun fibrous architectures through: (**d**) physical adsorption; (**e**) covalent immobilization; and (**f**) plasma treatment.

Generally, there are two distinct approaches through which genetic material can be loaded within electrospun fibrous architectures: (1) encapsulation into the interior of the fibers (i.e., blend electrospinning, coaxial electrospinning and emulsion electrospinning), or (2) immobilization on the surface of preformulated electrospun fibrous architecture (i.e., physical adsorption, covalent immobilization and plasma treatment) [75] (Figure 2).

The performance of the selected method in engineering electrospun fibrous architectures with optimal features to efficiently mediate gene therapy drug delivery and transfection is mainly impacted by the compatibility of the fibrous matrix and the loaded non-viral vectors, the interactions between the formulated system and tissue/cells as well as the targeted applications. The main strategies employed in engineering electrospun fibrous architectures with proper characteristics for controlled and sustained gene therapy drug delivery, along with the main advantages and shortcomings, are concisely described in the following section and are summarized in Table 3.

**Encapsulation approach**: Genetic material is loaded within the electrospun fibrous architecture during the electrospinning process by including it into the precursor system using blend, coaxial or emulsion electrospinning methods. The release of loaded cargo is generally driven by three factors: the compatibility between the genetic material and fibrous matrix, its diffusion abilities and the degradation rate of the electrospun fibrous matrix [75]. 

(*a*) *Blend electrospinning* implies the incorporation of genetic material within the nanofibrous matrix through simple mixing with the polymer solution, followed by electrospinning and the formulation of gene-loaded electrospun fibrous structures. By manipulating the physico-chemical characteristics of the electrospun architectures, the release kinetics of loaded genes can be controlled and modulated in accordance with the targeted application and expected therapeutic outcomes [32,83]. In 2003, Luu and co-workers [84] pioneered an electrospinning strategy to formulate polymer/DNA electrospun scaffolds for therapeutic gene delivery in tissue engineering. The electrospun membranous structures loaded with pDNA (encoding β-galactosidase), consisting predominantly of PLGA/PLA-PEG block copolymers, were capable of protecting the loaded pDNA and releasing it in a sustained manner that can be modulated by varying the copolymer ratio, and the integrity of the released pDNA was confirmed through efficient cellular transfection. Leveraging the same strategy, Nie et al. [85] proved that pDNA (encoding BMP-2)/chitosan (CS) nanoparticles may be encapsulated within PLGA/hydroxyapatite (HAp) composite nanofibrous scaffolds without affecting their biological activity or damaging cell activity while improving cell attachment and transfection in hMSC cells cultured in vitro, and Achille et al. [86] managed to efficiently deliver and release the bioactive RNAi-based pDNA from an electrospun PCL scaffold as a feasible route in suppressing cancer cell proliferation and in inducing cell death. In another work, Rujitanaroj et al. [87] combined the gene-silencing approach with the biomimicking features of nanofibers through constructing collagen type I (COL1A1) siRNA-encapsulated PCLEEP nanofibers for the effective long-term control of fibrous capsule formations. The ability of nanofiber constructs in reducing fibrous capsule thickness through the downregulation of COL1A1, along with the probability of delivering siCOL1A1 and cell-penetrating peptide (CPP) complexes for scaffold-mediated long-term gene silencing applications, was confirmed by the results of both in vitro and in vivo investigations. 

(*b*) *Coaxial electrospinning* employs two solutions (a polymer solution and a biological solution) which are concomitantly electrospun through different feeding capillary tubes in one needle, generating fibrous structures with core-shell architectures. Since the therapeutic genes are loaded in the core of the fibers, beside their high protection, these constructs assure a prolonged and sustained release profile that is characterized by a lowered burst effect as compared to other electrospinning strategies. Supplementarily, owing to the shell that acts as a diffusion barrier, these systems offer good control over the release kinetics of genes, which can be accurately modulated through adapting the shell structure [82]. For instance, by aiming to modulate the loading and release profile of a non-viral vector over an extended period of time, Saraf et al. [76] employed coaxial electrospinning to engineer core-sheath fibrous scaffolds, with the core consisting of aqueous solutions of PEG loaded with pDNA and PCL sheath encapsulating a PEI-HA (hyaluronic acid) non-viral gene delivery vector. The authors investigated the impact of the main parameters (e.g., PCL concentration, PEG concentration, molecular weight of PEG and concentration of plasmid DNA) on the morphological features of fibers, release kinetics and the transfection efficiency of formulated structures. It was observed that the modulation of each investigated parameter impacted the average diameter of the fibers, without significantly affecting the release rate of the investigated systems. It was also noted that the concentration of pDNA represented the main parameter driving the release kinetics, and, by properly tuning the coaxial fabrication parameters, the release profile of both components (pDNA and PEI-HA) could be coordinated, resulting in a persistent gene expression extended over 60 days in vitro. Later, aiming to improve the efficiency of cancer therapy, Sukumar et al. [88] reported, for the first time, the fabrication of a composite core-shell nanofibrous scaffold that can simultaneously deliver a suicide gene and a prodrug into cancerous cells. The PEO/bPEI electrospun nanofibrous scaffolds with core-shell architectures loaded with 5-Fluorocytosine (5-FC) within the core for controlled and sustained delivery and CD::UPRT polyplexes loaded into the shell were obtained through coaxial electrospinning. The authors investigated the ability of the scaffold to induce apoptosis by suicide gene therapy in A549 cancer cells at both phenotypic and genotypic levels. The results indicated that the efficient expression of the suicide gene by cancerous cells, along with the controlled and sustained release of prodrugs, triggered cellular apoptosis that also impacted the cells in the vicinity through bystander effects, drastically improving the anticancer efficacy of the dual delivery system. Using the same approach, Xie and co-workers [89] constructed a bioactive core-shell PEI/pBMP2-PLGA electrospun scaffold as a gene-activated matrix (GAM) for gene delivery, which was able to promote periodontal tissue regeneration. The osteogenic factor was encapsulated in the PEI core, and PLGA was used as a shell that could protect the pBMP2 from direct exposure to organic solvents and that could control its release. The formulated GAM was capable of assuring a continuous release of loaded cargo over a period of 28 days and an improved transfection efficiency along with a prolonged expression time of BMP2 (more than 28 days) after being cultured in vitro in hPDLSCs, as compared to standard electrospun counterparts.

Although a limited number of coaxial electrospun-fibrous-architecture-mediated gene therapy drug deliveries are reported so far, the capability of loading different bioactives in each layer makes the core-shell architectures potent substrate templates for tailoring the delivery kinetics of multiple gene vectors, thus broadening their applications in various fields of biomedicine [2].

(*c*) *Emulsion electrospinning* represents another method employed in the incorporation of genetic material within the electrospun structure with a core-sheath architecture, which is directly loaded into a spinning system consisting either of water-in-oil (W/O) or oil-in-water (O/W) emulsion stabilized by an emulsifier [81]. Considering the simplicity and versatility in the encapsulation of both hydrophilic and hydrophobic bioactives, this method has been widely explored in the construction of electrospun fibrous architectures with highly proper features for localized and controlled gene therapy drug delivery. For instance, targeting the gradual release of pDNA along with the promotion of tissue regeneration and wound recovery, Yang and co-workers [90,91] managed to design viruses mimicking core-sheath structured fibers based on PELA with PEI/pDNA(encoding pbFGF) polyplexes loaded into the core and with PEG included on the fiber sheath through the emulsion electrospinning method. The formulated pbFGF-loaded electrospun mats showed the significant ability to enhance cell proliferation as well as efficient cell transfection that was extended for over 28 days in mouse embryo fibroblasts cultured in vitro. Furthermore, the skin regeneration performances of pbFGF-loaded fibrous mats were validated in vivo on diabetic rats, confirming that the gradual release of DNA led to a substantially high wound recovery rate, accompanied by improved vascularization and completed tissue re-epithelialization. He et al. [92] applied the emulsion electrospinning strategy to formulate core-sheath electrospun fibers loaded with multiple pDNA polyplexes as prospective platforms towards the regeneration of mature blood vessels. The polyplexes (pVEGF and pbFGF) were loaded within the core, and PEG was incorporated into the fiber sheath to modulate the release of pDNA polyplexes from core-sheath architectures based on PELA. The in vitro investigations highlighted the capability of scaffolds to release the loaded cargoes in a sustained manner extended for ~4 weeks along with significantly diminishing the initial burst effect, promoting cell attachment and viability, cell transfection and protein expression, which enhanced the generation of microvessels and the formation of mature vessels and also alleviated the inflammation reaction. In another work, Cui et al. [93] constructed bilayered electrospun membranes dual-loaded with TPR/miR-126 and TPV/miR-145 complexes for the target regulation of VECs and VSMCs during small-diameter vascular regeneration. PELCL containing 10% PEG to accelerate the release of miR-126 for rapid endothelialization was used to formulate the inner fibers and, owing to its good mechanical properties as well as to prolonging the release of miR-145 PLGA, was employed in the outer one, resulting in the bilayered electrospun membranes with 1.5 mm diameters with nano-sized inner fibers and micron-sized outer diameters. The in vitro studies showed the fast cumulative release of miR-126 in the first 10 days, whereas the PLGA core sustained the release of miR-145 for about 56 days. Furthermore, the capacity of membranes to modulate VECs and VSMCs promoting rapid endothelialization and inhibiting VSMC hyperproliferation was validated by both in vitro and in vivo investigations, proving the feasibility of the proposed approach in tissue regeneration. Recently, Wen et al. [94] reported the construction of REDV-functionalized aqueous PELCL core-shell electrospun membranes loaded with microRNA-126 with suitable mechanical properties and prolonged cumulative release of loaded genetic material for the efficient modulation of vascular endothelial cells (VECs). 

**Immobilization approach**: The strategy involves the attachment of genetic material onto the surface of preformulated electrospun fibrous architectures via physical adsorption or covalent immobilization. This approach can prevent the loss of genetic material bioactivity that may occur during the formulation process (e.g., exposure to organic solvents or high electrical fields), and it may offer localized delivery directly to the targeted location within the cellular microenvironment, avoiding mass transfer issues [2,75]. 

(*d*) *Physical adsorption* is the simplest method for loading genetic material onto the electrospun fibrous architectures using non-covalent interactions (e.g., van der Waals, hydrophobic and electrostatic interactions), which is usually realized through immersing the nanofibers into the aqueous solution or emulsion-containing genes. Although the method does not impact the therapeutic activity of loaded cargoes and numerous studies have employed this approach in the design of optimal electrospun fibrous architectures for gene therapy drug delivery, the uncontrolled release profile still remains a critical disadvantage [5]. According to the literature, for the first time, Hu and co-workers [95,96] addressed the electrostatic interactions to immobilize PEI/DNA complexes onto nanofibrous scaffolds for in situ transfection applications. In this context, using a dual-jet system, the authors combined the contributions of two different polymers, the natural polycationic alginate and the synthetic PCL, into electrospun fibrous architectures, which, on one side, assures the necessary negative functionalities for the immobilization of positively charged PEI/DNA polyplexes and, on the other site, improves the biocompatibility and transfection rate. The in vitro experiments demonstrated the ability of composite Alg/PCL nanofibrous structures to successful deliver the loaded genes to targeted cells as well as improved in situ transfection activity that can be modulated through controlling the highly tunable physico-mechanical characteristics of scaffolds. Then, the authors investigated electrophoretic deposition as a feasible strategy for efficient gene immobilization and promoting transfection activity, demonstrating that, by applying a low-voltage direct-current electric field, the degree of immobilized genes, the degradation rate of alginate and in situ gene transfection efficiency may be controlled and increased, according to the targeted application [97]. 

(*e*) *Covalent immobilization* represents a complex method employed in the immobilization of genes on the surface of electrospun fibrous architectures via chemical bonds (e.g., peptide bonds). Considering that this method involves a proper covalent bonding/reaction that may generate non-uniform modifications on the nanofiber surfaces that may further alter the global properties of the materials and that the controlled release of loaded genes can be achieved in the presence of external enzymes, this method is not considered a regular route to formulate gene-loaded electrospun fibrous architectures [32]. Kim et al. [98,99] used this approach to engineer matrix metalloproteinase (MMP)-responsive PCL-PEG electrospun nanofibrous structures capable of releasing pDNA (encoding hEGF) in response to MMPs inherently overexpressed in diabetic ulcers in order to be applied as a local gene delivery system for skin tissue regeneration. The MMP-cleavable linker was conjugated to the amine group on the linear PEI which was presented on the surface of the fibrous matrices, and electrostatic interactions were used to anchor pDNA-hEGF. According to the in vitro investigations, localized and controlled gene expression was obtained that originated from the cleaved DNA-hEGF after its exposure to MMPs, increasing the expression levels of hEGF in primary human dermal fibroblasts (HDFs) as well as accelerating wound healing rates (burn recovery) in the animal model after 7 days of treatment. Using the same strategy, Monteiro and co-workers [100] combined liposome gene delivery with fibrous architectures to develop a multi-functionalized electrospun scaffold with potential applications in bone tissue regeneration through the covalent immobilization of pDNA (encoding RUNX2) loaded into liposomes on the surface of PCL-based nanofibrous mesh. Based on a comprehensive in vitro evaluation, it was demonstrated that this strategy can produce long-term gene expression along with the osteogenic differentiation of hBMSCs with the feasibility to be used in the enhancement of the osteoinductive properties of scaffolds used in bone tissue engineering. 

(*f*) *Plasma treatment* represents another feasible strategy employed for the chemical modification of electrospun fibrous architecture surfaces through the generation of various functionalities (e.g., amino, hydroxyl or carboxyl) after treating the surface of the nanofibers with several plasma gases, including ammonia, nitrogen, oxygen, carbon dioxide, argon and air [101]. This high-energy cost-effective treatment can be applied to modulate surface properties [102], enhance hydrophilicity [103], increase the absorption of bioactive molecules (proteins, drugs, growth factors or non-viral vectors) as well as to promote cellular adhesion and proliferation [104,105] on the surface of electrospun fibrous architectures in biological environments. Sultanova et al. [106] formulated nanofibrous scaffolds with core-shell architectures consisting of a PVA-chitosan core and a PCL shell as potent gene delivery systems. In order to increase the hydrophilicity of the fibers that further promote and improve cell adhesion, the surfaces of the electrospun fibers were subjected to plasma treatment in the presence of O_2_, whereas the significantly improved surface hydrophilicity of the treated fibers was confirmed by contact angle measurements. The authors envisaged further investigations regarding plasmid loading into chitosan, gene release profiles as well as gene transfection efficiency to examine the usefulness of the bioactive scaffolds for gene delivery. In another work, Tahmasebi and co-workers [103] inserted two important miRNAs (miR-22 and miR-126) into PCL electrospun nanofibers, aiming to develop fibrous scaffolds that, beside their fibrous topography, may play a regulatory role during osteogenesis, improving the osteogenic differentiation of human-induced pluripotent stem cells. After morphological characterization, which showed that gene incorporation did not impact the size and morphology of the scaffold, a detailed biological investigation was conducted by evaluating protein absorption, cell adhesion as well as osteoconductivity through assessing the osteogenic differentiation potential of induced pluripotent stem cells (iPSCs). Particularly, improved protein absorption and cellular attachment abilities were noted in the case of the miRNA-inserted scaffolds when compared to other groups. To better understand this behavior, the authors exposed the formulated materials to plasma treatment, observing a substantial decrease in surface hydrophobicity after both miRNA loading and plasma treatment, thus explaining the increased cells and protein absorption. Furthermore, bone-related genes and protein expression assays highlighted the highest osteogenic markers in iPSCs grown on the miRNA-incorporated fibers compared to the cells cultured with standard PCL counterparts, proving their great potential in the regeneration of bone lesions and defects. Recently, aiming to develop more efficient carriers for local and controlled gene delivery, Malek-Khatabi et al. [107] combined microfluidics with electrospinning technology and constructed a smart and environmentally responsive gene delivery platform for the repair and regeneration of bone tissue. To this end, microfluidic-assisted synthetized nanocomplexes consisting of CS/pDNA encoding BMP-2 were conjugated through sensitive disulfide bonds to metalloprotease (MMP)-sensitive peptide sequences. Prior to conjugation, MMP-peptides were immobilized on the surface of PCL electrospun scaffolds by EDC/NHS chemistry after the oxygen plasma treatment of fibers, followed by controlled hydrolysis to increase the number of carboxylic groups on the nanofiber surfaces. The in vitro and in vivo investigations proved the ability of smart and environmentally sensitive scaffolds to promote the osteogenic differentiation of stem cells.

## 4. Electrospun-Fibrous-Architecture-Mediated Gene Therapy Drug Delivery

It is well-known that the main criteria of scaffold-mediated gene delivery in tissue engineering and regenerative medicine, beside biocompatibility and the capacity to support gene transfection, are the abilities to promote and maintain the phenotype and to guide the development of new tissue, mimicking the native mechanical and biochemical features [16]. Biodegradability and bioresorbability are among other primordial characteristics of materials used in regenerative medicine that drive material integration with surrounding tissue, avoiding inflammatory/immune responses that may lead to scaffold rejection and/or tissue necrosis [108]. In the frame of the current challenges, a large variety of naturally derived or chemically synthetized polymers in different conformations and compositions were explored in the construction of electrospun fibrous scaffolds with highly proper characteristics for the optimal guidance of gene therapy drug delivery, thus improving therapeutic outcomes and promoting clinical translation. Therefore, according to the nature and composition of the spinning system, the electrospun fibrous architectures for controlled gene therapy drug delivery can be classified into natural, synthetic and natural/synthetic polymer-based scaffolds. The main biological/therapeutic performances of electrospun fibrous architectures as gene therapy delivery systems in the context of the above-mentioned classification are discussed in the following section and are also summarized in Table 4.

### 4.1. Natural Polymer-Based Electrospun-Fibrous-Architecture-Mediated Gene Therapy Drug Delivery

Derived from their transient nature, natural polymers, although characterized by insufficient stability (fast degradation rate) and low mechanical properties, have features such as versatility in formulation, high biocompatibility, biodegradability and biomimetic features that promote cell proliferation and differentiation, which make them potent candidates in engineering gene-loaded scaffolds with nanofibrous architectures that are feasible for biomedical applications [16,121]. Among the variety of naturally occurring polymers, proteins and polysaccharides are the most employed biomaterials in electrospinning and the production of nanofibrous architectures for the delivery of therapeutic genetic material. For instance, Karthikeyan et al. [122] reported for the first time the delivery of siRNA through zein nanofibers, investigating the efficiency of these protein-based fibers in protecting the integrity and sustaining the release of loaded therapeutic genes for their applications in biomedical fields. Based on comprehensive in vitro investigations, the authors proved the contribution of protein fibers in preserving the integrity of siRNA (gel agarose analyses) through physical protection and their ability to ensure the sustained release and a sufficient amount of loaded genes to induce the gene silencing effect (release studies) as well as to significantly promote the transfection of siRNA into the cells, thus improving the therapeutic efficiency of gene therapy. Aiming to formulate a controlled and sustained gene delivery system that is able to accelerate skin wound healing, one of the most complex biological processes in mammals, He et al. [109] developed a biodegradable nanofibrous gene-activated matrix (GAM) by immobilizing non-viral vectors consisting of DNA (pVEGF)-loaded PLGA/PEI nanoparticles modified with cell-penetrating peptide KALA onto a polydopamine-coated electrospun alginate nanofibrous scaffold. Initially, the authors conducted a series of in vitro analyses with respect to DNA-releasing behavior, degradation properties, transfection efficiency and VEGF protein expression, confirming that the 3D GAM is capable of not only assuring a prolonged and sustained release of the incorporated gene but also encouraging cell adhesion and sustained gene expression. The great potential of the designed material for mediating long-term cell functions and accelerating skin wound healing was further validated by the results of in vivo investigations that highlighted the remarkable wound-healing performance of the GAM system through its ability to promote complete re-epithelization, to reduce inflammatory responses and to increase neovascularization after 21 days of implantation into a full-thickness excisional skin wound model in rats. Pankongadisak and co-workers [110] proposed a new approach for polyplex delivery in regenerative medicine, engineering bioactive scaffolds with functional gene expression systems by combining the advantages of electrospun gelatin scaffolds with those of polyplexes. Polyplexes, including poly(aspartic acid) (pAsp), were generated by condensing pDNA into lipid-modified PEI that was further subjected to electrospinning, being uniformly embedded within the protein or protein-PEG solutions. It was noted that there was a significantly improved transfection activity of the complexes both in the solution and after entrapment in the electrospun mats in human myoblasts (C2C12) and mouse osteoblasts (MC3T3-E1) in vitro cultured cells, and it was attributed to the presence of anionic pAsp as well as to PEG addition in some concentration that improved the pDNA entrapment. Then, the electrospun mats loaded with BMP-2 polyplexes showed a fruitful effect on osteogenic differentiation behavior, being able to induce a robust osteogenic differentiation in both types of cells after 7 days of treatment, validating the feasibility and efficiency of the fibrous architecture in bearing bioactive pDNA complexes for the regenerative repair of tissues and their translation to in vivo investigations. 

In another work, James et al. [123] combined the unique ECM mimicking nanofiber scaffolds with their controlled delivery abilities and formulating gelatin nanofibrous scaffolds capable of the localized transient delivery of miR-29a inhibitors to increase ECM deposition in bone regeneration. The miR-29a-loaded electrospun gelatin nanofiber scaffolds were obtained through blend electrospinning, and the feasibility of scaffolds to deliver in a sustained and bioactive manner the encapsulated gene as well as the effectiveness of the scaffold to enhance EMC synthesis in cells via localized miR-29a inhibitor delivery were comprehensively evaluated in vitro. The release experiments showed the continuous release of miRNA inhibitors, which was extended over 72 h, and biological investigations performed on MC3T3-E1 cells or primary bone marrow stromal cells seeded on gene-loaded nanofibers displayed the production of more osteonectin along with increased levels of Igf1 and Tgfb1 mRNA, indicating efficient inhibitor delivery as well as collagen production.

### 4.2. Synthetic Polymer-Based Electrospun-Fibrous-Architecture-Mediated Gene Therapy Drug Delivery

Unlike naturally derived materials, the unique characteristics of chemically synthetized polymers, i.e., high versatility and reproducibility, total control over morphological, chemical and mechanical features, the precise tuning of the degradation rate and surface functionalities according to the application, make them indispensable and predominantly used materials in the construction of scaffolds for mediating gene delivery [124,125]. However, the lack of innate cell binding sites that hinder suitable cellular interactions, along with occasional cytotoxicity and immunogenicity issues, are the main drawbacks facing the in vivo application of these materials. Among the variety of synthetic materials, the FDA-approved poly (lactic-co-glycolic acid) (PLGA), poly(lactic acid) (PLA) and polycaprolactone (PCL) are the most exploited polymers in the formulation of gene-activated scaffolds with nanofibrous architectures, owing to their biocompatibility, biodegradability and multifarious design [126,127,128]. 

PLGA is a synthetic polyester, and the copolymer of poly-lactic acid (PLA) and poly-glycolic acid (PGA) has become ubiquitous in various formulations, with applications in different fields of biomedicine, remaining the best-defined biomaterial available as a drug delivery carrier with respect to design and performance. Beside its intrinsic biocompatibility, the resulting by-products (its original monomers) following its easy hydrolyzation in vivo are also biocompatible and are physiologically metabolized by the tricarboxylic acid cycle for final excretion in the lungs [129]. Therefore, electrospun nanofibrous scaffolds based on PLGA loaded with DNA encapsulated into core-shell micelles based on the triblock copolymer of poly(lactide)-poly(ethylene glycol)-poly(lactide) were engineered and investigated as gene vehicles that may preserve the integrity and bioactivity of DNA as well as control the release kinetics and transfection efficacy through the degradation rate of the scaffolds [85]. In another work, pDNA/dendrimer complexes were immobilized onto layer-by-layer polyelectrolyte-coated electrospun PLGA nanofibers; the formulated systems were capable not only of supporting the attachment and growth of hMSCs but also of increasing cell differentiation towards the osteoblastic lineage in vitro [130]. Later, following the same strategy, Xiao et al. [131] managed to formulate biocompatible pDNA/G5·NH2 dendrimer-grafted electrospun PLGA nanofibrous mats by combining the layer-by-layer strategy with dendrimer chemistry. The grafted G5·NH2 dendrimer significantly increased the surface hydrophilicity of PLGA nanofibers, which showed good potency for efficiently complex pDNA and the in situ transfection of NIH 3T3 via solid-phase transfection. Aiming to improve gene transfection efficiency into stem cells, Wang et al. [132] embedded the ultra-strong adsorption capability and high surface area of GO with the nanofibrous architecture of PLGA to develop a biocompatible functional electrospun nanofibrous mat capable of delivering the PEI/pDNA to the stem cells, simultaneously acting as a substrate for their growth and differentiation. The authors hypostatized that the immobilization of PEI/pDNA onto the electrospun scaffold may facilitate the direct contact of cells and pDNA, promoting its entry into the stem cells and thus enhancing the gene transfection efficiency. Beside having good biocompatibility and a high amount of PEI/pDNA immobilized on the surface, the proposed system showed significant enhancement in DNA transfection efficacy in 293 cells and hMSCs cultured in vitro, proving their effectiveness in gene therapy and regeneration medicine.

The bioresorbable polyester PLA is another synthetic polymer with a wide spectrum of applications in tissue engineering for the function restoration of impaired tissues, owing to its good mechanical properties and processability [133]. Considering the chiral nature of the lactic acid molecule, several polymers are included in the PLA family, i.e., pure poly-L-lactic acid (PLLA), pure poly-D-lactic acid (PDLA) and poly-D,L-lactic acid (PDLLA) [134]. Since the slow degradation rate and intrinsic hydrophobicity represent the main shortcomings usually associated with PLA use, amphiphilic block copolymer poly(DL-lactide)-poly-(ethylene glycol) (PELA) is frequently preferred in the design of scaffolds with fibrous architectures and features that are important to tissue engineering. In the context of searching for new strategies that may tackle the challenges associated with the outcomes of vascular grafting (e.g., fully vascularizing engineered tissues), electrospun PELA scaffolds with core-shell architectures loaded with multiple polyplexes [92] or fibers loaded with multiple pDNA calcium phosphate nanoparticles [135] have been proposed as multiple gene delivery platforms capable of guaranteeing sustained and localized pDNA delivery for the efficient promotion of blood vessel generation, alleviating cytotoxicity and inflammatory responses. Recently, aiming to drive a pro-angiogenic response, a key-factor in efficient tissue repair and regeneration, Li et al. [112] constructed PLLA/POSS nanofibers loaded with pDNA (encoding pAng) condensed into N-trimethylchitosan chloride nanoparticles (TMC). The dispersion of functional polyhedral oligomeric silsesquioxane (POSS) nanoparticles generated a porous fibrous structure with improved biocompatibility and toughness, and the assembly of pDNA with TMC significantly enhanced gene stability and biocompatibility, which collectively led to a controlled and sustained release of pAng extended over 35 days along with higher and prolonged gene transfection efficiency in vitro. Furthermore, the ability of the scaffolds to efficiently promote angiogenesis and dermal wound healing was investigated in vivo using a full-thickness skin wound model, which highlighted that, after 3 weeks of implantation, the PLLA/POSS/pAng scaffolds presented better vascularization capability, prominently increasing the number of newly formed blood vessels and lumen areas as compared to other groups. In another work, Muniyandi et al. [136] demonstrated the capacity of a dual miRNA scaffold system to transdifferentiate cardiac fibroblasts by immobilizing muscle-specific microRNA (PEI-miR-1 and PEI-miR-133a) polyplexes on the fibronectin modified PLLA fibers. The in vitro studies revealed the ability of the scaffolds to enhance the reprograming efficiency of adult human cardiac fibroblasts into cardiomyocyte-like cells, in particular from the perspective of their translation into in vivo investigations patched with sustained gene delivery, targeting cardiac fibroblasts and regenerating the infracted heart. 

PCL, a hydrophobic aliphatic polyester with a semicrystalline structure consisting of hexanoate repeat units [137], is also extensively investigated in the biomaterial field, owing to its amenable mechanical properties and degradation kinetics that are longer as compared to PLGA or PLA [138]. Considering that, beside the above-mentioned advantages, PCL nanofibers can be aligned to mimic the ECM structure, this biodegradable polymer is of particular importance in the construction of electrospun fibrous architectures with optimal features for sustained non-viral gene delivery, capable of assuring improved gene transfection efficacy according to the targeted biomedical application. To date, electrospun scaffolds based on PCL with different structural, conformational and morphological features have been engineered and investigated as compelling strategies for the effective delivery of a wide range of genetic material (e.g., pDNA, siRNA or miRNA), showing the remarkable capacity to sustain and modulate the transfection properties of the loaded gene and assuring the optimal therapeutic outcomes in a particular clinical application, i.e., tissue engineering and regeneration [76,139,140] and central nervous system therapy [141,142]. Aiming to develop a new strategy for improving wound repair as an alternative to viral vectors that can be easily translated into clinical use, Kobsa et al. [143] managed to develop a multi-functional construct by integrating PLA/PCL into an electrospun scaffold capable of the highly efficient delivery of non-viral DNA (encoding keratinocyte growth factor (KGF)) to the wound, supplementarily supporting the intrinsic ability of the skin to heal. Based on a series of in vivo studies, it was demonstrated that the KGF plasmid-loaded scaffold may act as a bioactive substrate for the treatment of cutaneous wounds by significantly improving wound re-epithelialization, keratinocyte proliferation and granulation responses. Keeping in mind the goal of tackling the disadvantages associated with small interfering siRNA delivery (e.g., poor cellular uptake or short half-life) and improving its intracellular delivery, Pinese and co-workers [111] developed and investigated the feasibility of new fiber constructs for the controlled and localized delivery of siRNA/MSN-PEI complexes. Initially, to modulate and improve the gene loading efficiency as well as to offer control over target-selective delivery and cellular uptake, siRNA was complexed with PEI-modified mesoporous silica nanoparticles (MSN-PEI), resulting in siRNA/MSN-PEI complexes, which were further loaded within poly (caprolactone-co-ethyl ethylene phosphate) (PCLEEP) nanofibers or were immobilized onto the PCL fiber surface. The in vitro studies underlined the release of siRNA in a prolonged manner, extended over 30 days for siRNA/MSN-PEI-coated scaffolds and over 5 months in the case of siRNA/MSN-PEI-encapsulated scaffolds, along with improved siRNA transfection efficiency and sustained gene silencing in the targeted protein. The result of the in vivo subcutaneous implantation analyses showed a reduction in the fibrous capsule, with 45.8% in the presence of scaffolds as compared to negative siRNA after 4 weeks of treatment, demonstrating the biofunctionality and efficacy of the scaffold-mediated sustained delivery of siRNA/MSN-PEI for long-term non-viral gene silencing applications. Later, aiming to address the issues related to restenosis, which still remains the main challenge in small-diameter vascular regeneration, Wen et al. [114] synthetized VAPG peptide-modified PEG-trimethyl chitosan (TPV)-condensing miRNA-145, and the formulated TPV/miRNA-145 complexes were loaded in the poly(ethylene glycol)-b-poly(L-lactide-co-ε-caprolactone) PELCL electrospun membranes for the efficient modulation of vascular smooth muscle cells (SMCs). Although there was no evidence of in vivo investigations, according to the results of in vitro testing, the functional electrospun membranes were capable of controlling the SMCs at the gene and protein level, whereas the released miRNA-145 presented prolonged bioactivity in regulating the contractile phenotypes of SMCs that were extended over 56 days, proving their ability in the modulation of SMC phenotypes and proliferation during vascular regeneration.

### 4.3. Natural/Synthetic Polymer-Based Electrospun-Fibrous-Architecture-Mediated Gene Therapy Drug Delivery

Electrospun scaffolds embedding both natural and synthetic polymers represent the latest strategy in engineering fibrous-architecture-mediated gene therapy drug delivery capable of assuring efficient gene transfection and optimal therapeutic outcomes. Considering that this design principle allows high versatility in the construction of fibrous architectures with physico-chemical, morphological and biological features that can be easily modulated to better suit the mechanical, biological and physical demand of the targeted host tissue, this thriving approach is of particular importance to regenerative medicine. Therefore, numerous blends consisting of synthetic and natural polymers in different formulations (e.g., PCL/alginate [96], PLLA/Collagen [118], PCL/silk fibroin [120], Gelatin/Collagen/PEG [144], PLLA/silk fibroin [145]) were constructed and investigated as electrospun fibrous strategies for the efficient delivery of genetic material. For instance, Zhao et al. [118] managed to substantially improve transfection efficiency and rhBMP-2 transgene mRNA expression as well as to induce ectopic bone formation both in vitro and in vivo by adsorbing rhBMP-2/p-DNA complexes onto PLLA/Collagen I electrospun scaffolds. Zhou’s group [119] constructed a bilayer vascular scaffold consisting of a PELCL inner layer loaded with polyplex/miRNA-126 complexes and a PCL/gelatin outer layer for the efficient intracellular delivery of miR-126 to vascular endothelial cells. The scaffold showed a great ability to load (68%) and then release in a sustained manner (extended over 56 days) the therapeutic miR-126 complexes, along with high potency to modulate VEC proliferation via the down-regulation of SPRED-1 gene expression in vitro. Then, the in vivo assessment suggested that the electrospun vascular grafts could enhance re-endothelialization after 8 weeks of replacing the rabbit carotid artery, proving its potential as a platform for the localized delivery of miRNAs to facilitate blood vessel regeneration. Liu and co-workers [146] constructed and investigated a multi-functional and structurally simple gene carrier based on 2,6-pyridinedicarboxaldehyde-polyethylenimine (PDA)-mediated extracellular signal-regulated kinase (ERK)2-siRNA carrier, electrospun into poly-l-lactic acid/hyaluronan (P/H) fibers with the ability to perform the sustained release of bioactive siRNA for the long-term prevention of adhesions and ERK2 silencing in peritendinous anti-adhesion applications. The authors hypothesized that the good capacity of the materials in constantly blocking ERK2 and its downstream SMAD3 signal to accomplish tissue separation and prevent adhesion formation may originate from the ability of ERK2-siRNA-loaded PDA electrospun fibrous membranes to prevent the adhesions and assure a long-lasting release of bioactive siRNA along with the protection of loaded ERK2-siRNA by the cationic PDA carrier. In another work, Mulholland et al. [147] approached a new angiogenic therapy that may reduce endogenous FKBPL levels and promote angiogenesis for advanced wound healing, using an amphipathic peptide (RALA) to condense siFKBPL, resulting in RALA/siFKBPL complexes, which were further incorporated into a bilayer electrospun nanofibrous patch based on alginate/PVA and Chitosan/PVA. The proposed therapy determined the enhancement of cell migration and the tubular-forming capacity of HMEC-1 cells cultured in vitro, along with a significant increase in angiogenesis compared to controls, which were tested in vivo in a full-thickness skin wounding model in C57BL/6J mice. Recently, instead of classical mono-layer mats for gene delivery applications, Tsekoura and co-workers [144] took a different approach by constructing a two-layered mat consisting of collagen fibers in the first layer and a mixture of gelatin, collagen and PEG-embedding pDNA complexes in the second layer, which showed a great ability to increase the transgene expression and induce osteogenic activity in vitro, sustaining their potential for further in vivo/clinical investigations.

## 5. Recent Advances of Electrospun Fibrous Architectures as Amenable Strategies for Gene Therapy Drug Delivery

**Figure 3:**
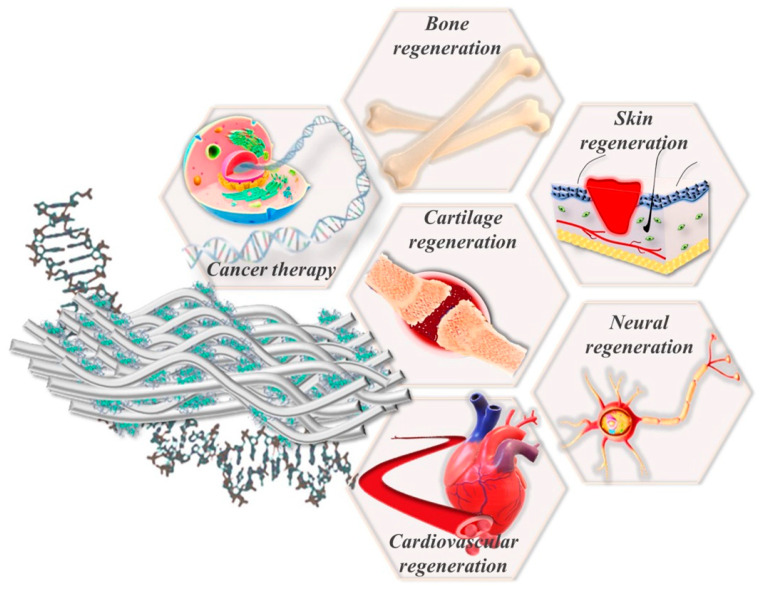
Schematic representation of the main applications of electrospun-fibrous-architecture-mediated gene therapy drug delivery in tissue regeneration and cancer therapy. The nucleic-acid-encoding therapeutic gene may be loaded per se or may be complexed with non-viral vectors.

Since different electrospun fibrous architectures loaded with diverse bioactive molecules can be easily engineered through selecting proper materials and approaches, this fibrous-architecture-mediated gene therapy drug delivery, beside its ECM-analog nature, offers huge versatility in the modulation of topography, in gene loading degrees and in release kinetics to potentially meet the required characteristics and assure the optimal therapeutic outcomes in a particular clinical problem/need [82]. Some of the latest therapeutic advances of gene-loaded electrospun fibrous architectures used in tissue engineering and regenerative medicine are discussed in the following section, and an overview with the most important applications of electrospun-fibrous-architecture-mediated gene therapy drug delivery in different fields of biomedicine is presented in Figure 3. 

*Wound healing and skin tissue regeneration*: Skin tissue engineering has emerged as a potent means capable of promoting wound healing, a complex and dynamic regenerative process that can be achieved by effectively coordinating hemostasis, inflammation, epithelization, angiogenesis and remodeling/maturation [2,82]. Since wound healing is an interactive process controlled by temporal interactions between different dermal cells, extracellular matrix components and signaling molecules, the development of cost-efficient and reproductible strategies with highly proper features to efficiently improve wound healing and to promote its clinical translation remains a challenge to the scientific community, particularly due to the complexity of the underlying biological processes [143]. Therefore, the high porosity and surface area, along with the ability to precisely mimic the morphology of ECM and its versatility in functionalization, support the electrospun fibrous architectures as optimal and robust templates for promoting skin tissue regeneration, satisfying the goal of conventional wound dressings [82,148]. To date, standard/multi-functional electrospun fibrous architectures consisting of different polymers and loaded with a wide range of genetic material have been formulated and extensively investigated as templates with proper biochemical and physicochemical characteristics for mediation in gene therapy drug delivery, and they guide cellular behavior, ensuring the optimal therapeutic outcomes in wound healing and skin tissue regeneration applications. Recently, Mulholland et al. [116] demonstrated for the first time the potency of CHAT/pmiR-31 nanoparticle-loaded electrospun PVA wound patches as a viable therapy for effective wound repair. The efficient delivery of pmiR-31 was promoted by complexation with CHAT peptides, resulting in nanocomplexes with high cellular uptake as well as improved miR-31 expression, transfection efficiency and angiogenesis potential in both skin human keratinocytes (HaCaT) and human microvascular endothelial cells (HMEC-1) cultured in vitro. In vivo assessment of a full-thickness skin wounding model in C57BL/6 J mice demonstrated that the CHAT/pmiR-31-loaded wound patch significantly enhanced keratinocyte functionality as well angiogenesis compared to untreated or commercial controls, proving its enhanced wound repair potency through both epithelial and stromal compartments. 

*Central nervous system tissue regeneration*: Considering that nerves present a lesser ability for regeneration after traumatic injury, innovative and proper approaches capable of inducing functional repair and neural regeneration are crucially necessary [149]. Owing to its high feasibility in modifying orientation and topographical features, the application of gene-loaded electrospun scaffolds are thriving, and versatile strategies that are able to guide neuronal cell growth or neuronal differentiation on their surfaces in both peripheral nerve injuries and central nervous system (CNS) injuries [149,150]. Myelination represents one of the crucial processes involved in functional recovery that guide the rapid sensory-motor coordination in vertebrates and is managed by the oligodendrocytes (OL), the myelinating cells of the CNS. In this respect, Ong et al. [151] reported a biofunctionalized microfiber scaffold consisting of suspended-axon-mimicking PCL-based fibers loaded with a cocktail of microRNA (miR-219 and miR-338) as a microfiber platform for the controlled and sustained delivery of promyelinogenic biomolecules to oligodendrocyte precursor cells, being capable of promoting their in vitro differentiation and myelination as well as enabling the screening of therapeutics for remyelination. Following the same challenge, Nejati et al. [152] proved that combinatorial therapy can offer effective repair for spinal cord injury (SCI) axonal regeneration in vivo through constructing a biodegradable emu oil-loaded PCL/Col electrospun nanofibrous scaffold seeded on the surface with adipose-derived stem cells (ASCs) genetically engineered to over-express GDNF. Beside glial scar formation, enhancement was noted in the release of neurotrophic factors that provide a favorable microenvironment to enhance axonal regeneration and to improve the locomotor recovery in rats after 8 weeks of scaffolds post-transplantation. An important contribution in the advancement of spinal cord (SC) and CNS regeneration is the work performed by Chew and co-workers [153,154,155], who managed to promote OPC differentiation, axon regeneration and CNS remyelination in vivo through a hybrid scaffolding system consisting of core bundle aligned PCLEEP fibers structurally stabilized in a 3D conformation using collagen hydrogel encapsulating different cocktail of miRNAs, which were capable of assuring the localized and sustained delivery of promyelinogenic biomolecules within the injured CNS as well as of enhancing axion regeneration in SC injuries. Based on a complex in vivo investigation, the authors demonstrated that the proposed fiber-hydrogel scaffold can enhance the number of Olig^2+^ oligodendroglial lineage cells, differentiate OLs and remyelination and significantly promote functional recovery after post-treatment in rats. Recently, inspired by the acidic micro-environment at acute injury sites, Xi et al. [156] reported the construction of a functional immune micro-environment responsive fiber scaffold as a strategy for the repair of acute SCI. IL-4 plasmid (pDNA)-loaded cationic liposome-modified aldehyde functionalities were immobilized on the surface of amino-modified oriented electrospun HA-PLA core-shell fiber scaffolds through acidic-sensitive linkers. Under acidic conditions, there was an increase in the expression of anti-inflammatory factors along with a downshift in the level of pro-inflammatory factors in macrophages cultured in vitro in the presence of fiber grafted with liposomes carrying pDNA. Then, the fiber scaffold loaded with liposomes and NGF was implanted into a rat spinal cord injury model, noting an increased level of anti-inflammatory factors, accompanied by a decreased pro-inflammatory level and improved functional recovery after 8 weeks of treatment.

*Vascular tissue regeneration*: It is well-known that both endothelial and smooth muscle cells from the surface of vessel architectures, along with angiogenic growth factors, physical guides and supports, are among the crucial elements involved in vascular tissue regeneration [2]. Hence, electrospun-fibrous-architecture-mediated gene therapy drug delivery, which, beside ECM resemblance, may behave as supportive for endothelial cell development and may provide efficient physical guidance for new tissue, is of particular importance to vascular tissue regeneration, being widely explored in different applications. In this regard, Zhou et al. [157] reported a dual-functional electrospun membrane capable of accelerating vascular tissue regeneration by combining Arg-Glu-Asp-Val (REDV) peptide-modification of the surface of PELCL fiber to improve vascular endothelial cell adhesion as well as the encapsulation efficacy of miRNA-126 complexes. The formulated electrospun membranes presented modulated and local release kinetics of complexes along with the significant improvement of vascular endothelial cell adhesion and proliferation, proving their potency in the guidance and modulation of vascular tissue regeneration.

*Cancer therapy*: Since cancer represents a complex process originating from both genetic and epigenetic alterations, as well as from molecular and signaling pathway alterations occurring in tumor cells [158], the rational combination of electrospun fibrous architectures with chemotherapeutics and/or gene therapy drug delivery may represent a potent solution capable of enduring challenges related to insufficient therapeutic activity and drug or multi-drug resistance through assuring localized and significantly improved pharmacokinetic profiles of cargoes, increasing bioavailability and therapeutic outcomes with minimal side effects. A variety of chemotherapeutic agents, e.g., small molecular drugs, aptamers and nuclei acids, can be incorporated into electrospun fibrous architectures and can then locally release through diffusion-controlled or degradation-controlled release profiles depending on the materials and formulation strategies [82], which further can be used as standalone or as combinatorial chemotherapy. Aiming to mitigate issues related to the safety and efficacy of chemotherapy, Al-Attar et al. [159] investigated a novel combination of drug delivery devices composed of holo-transferrin-conjugated liposomes for siRNA delivery and electrospun PCL-GT microfibers for resveratrol release, which may generate an additive or synergistic overall effect originating from the presence of both therapeutics, which targeted different cellular pathways. It was noted that the strategy of combining two different delivery devices impacted cancer treatment to a greater extend by significantly affecting cell apoptosis in co-cultures of HUVECs and K562, inducing a 92.7% non-viability after 8 days of in vitro incubation. Recently, Jindal et al. [160] constructed a core-shell nanofibre scaffold capable of mediating the transfection of the connexin-43 gene into Cx43-deficient breast cancer cells (MCF-7), followed by the controlled and sustained release of 4-PB (a histone deacetylase inhibitor), showing great anti-proliferative potential activity in vitro and huge therapeutic translation ability via the Cx-based improvement of anti-cancer potential.

*Other applications*: Jin and co-workers [161] proposed a new complex co-delivery construct that was able to allow rapid ECM responses in soft tissue regeneration by loading lysyl oxidase-like 1 plasmids (pLOXL1) into nanoliposomes through microfluidics, which were further encapsulated into the core layer of the core-shell nanofibers through microsol-electrospinning. In vivo experiments that were performed on a rabbit model of abdominal hernia confirmed the ability of “patch” to accelerate local EMC reconstruction as well as the feasibility as a new concept in the development of new pelvic floor biomaterials and treatment methods for pelvic organ prolapse. In another work, Cai et al. [162] proposed an anti-adhesion hydrogel-electrospun fiber bilayer patch as an on-demand and unidirectional delivery strategy for peritendinous anti-adhesion. The in vitro investigations showed the MMP-2-responsive and unidirectional release behaviors of encapsulated TGF-β1 siRNA polyplexes from the proposed design along with associated gene silencing effects on TGF-β1, which generated the inhibition of fibroblast proliferation. The anti-adhesion potential was further validated in vivo by wrapping the repaired tendons of rats with the composite patch and by noting that, after 21 days of treatment, the peritendinous adhesion formation was successfully attenuated, and the side effect of TGF-β1 siRNA on the mechanical strength of the repaired tendon was reduced. Zheng et al. [163] demonstrated the effectiveness of combining photothermal and gene delivery strategies into electrospun nanofibers in localized gene transfection by constructing a photothermally activated core-sheath PLA-gelatin-based electrospun hybrid nanofiber encapsulating pDNA (encoding bFGF) in the core and gold nanorods with photothermal properties in the fiber sheath. The nanofiber mat presented a remarkable and highly tunable photothermal response under NIR irradiation, accelerating the release of pDNA and improving the transfection activity in NIH-3T3 fibroblasts as well as promoting the proliferation and migration of the transfected cells in vitro, suggesting their great potential in tissue engineering and cell-based therapy.

## 6. Conclusions, Challenges and Perspectives

**Figure 4:**
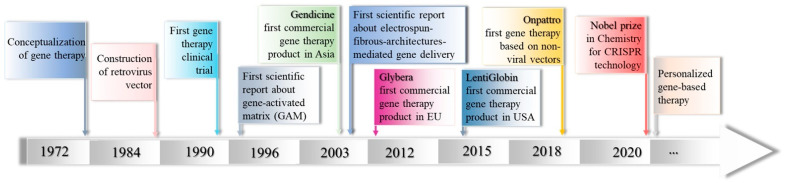
Timeline overview of key events for gene-based therapy.

Gene therapy has rapidly become a promising alternative to conventional therapeutic strategies, owing to its broad prospect and huge potential in offering long-lasting and curative benefits in the treatment of a multitude of human-inherited and acquired ailments. Its amplified interest, rapid evolution and extensive applications in both academic and clinical practices are reflected in the large amounts of basic and clinical research reported in the literature, enormous financial support that has been invested in the advancement of gene therapy and the improvement of innovative vectors as well as state-of-the-art gene therapy drug products that have been resoundingly approved by the FDA (Figure 4) [1,40]. 

Currently, there are approximately 23 gene therapy drug products approved for the treatment of human diseases worldwide [164], and the market for gene therapy is estimated to reach 13.0 billion USD by 2024 from 3.8 billion in 2019 [165]. Although viral and non-viral vectors represent basic gene therapy carriers, their widespread clinical applications are still controversial with respect to safety, efficiency, sustainability and stability in gene expression, biodistribution and pharmacokinetics as well as scalability [166]. In this context, the development of highly potent gene therapy drug delivery systems that are able to tackle the inherent challenges of gene therapy, particularly those related to retaining delivery efficacy and minimizing unwanted immune responses, has always been regarded as an utmost challenge that must be debriefed for further advances in gene therapy drug delivery technologies.

Electrospun fibrous architectures undoubtedly have emerged as highly promising and multi-functional strategies capable of improving gene therapy drug delivery for a wide range of biomedical applications. The high diversity in source materials, versatility in the formulation and encapsulation of various genetic material along with high amenability in modulating morphological and physicochemical features and biological performances of electrospun fibrous architectures as efficient gene therapy drug delivery mediators have contributed to widespread implementations [6]. The rationale behind the incorporation of genetic materials within these architectures is to provide gene protection and efficient, controlled and localized delivery in a spatio-temporal manner to the targeted site of action. 

Nevertheless, there is still a large gap between the laboratory validation of clinical investigations/trials and actual commercialization on the pharmaceutical market, considering that no evidence of electrospun products for gene therapy drug delivery are approved by the FDA so far. Apparently, even if substantial in vitro and in vivo work has been performed to investigate the behavior of electrospun-fibrous-architecture-mediated gene therapy drug delivery in complex biological environments, the differences in the complexity and metabolism systems between animals and humans may determine unexpected results during clinical trials and therapeutic outcomes [82]. Furthermore, challenges related to morphology and the structural and mechanical aspects of electrospun fibrous architectures capable of accurately mimicking the spatial and temporal control of the expression of multiple genes in tissues and organs should also be addressed [2]. Moreover, issues related to economic aspects in the production of electrospun fibrous architecture, including scalability and high reproducibility, still demand enormous efforts to be solved, considering the low yield of laboratory electrospinning equipment [82]. The studies concerning the long-term effects of processing parameters (e.g., charges accumulation), optimal designs and the compatibility of multi-component electrospun structure therapeutic genes or complex therapeutic system cells/tissue after implantation are still in their infancy, hindering the translation of scientific/academic results into clinical applications. In this respect, the advancement of complex/multi-functional therapeutic platforms capable of efficiently delivering gene therapy drugs to specific organs or tissues can possibly comprise defined designs in combination with complexation strategies that endow predictable pharmacokinetic and pharmacodynamic features as well as well-understood intracellular mechanisms of action and cellular response/behavior [8]. Despite the barriers that remain, electrospun-fibrous-architecture-mediated gene therapy drug delivery represents a broad and potent strategy used in various field of biomedicine, and multidisciplinary research progress along with technological advancements promote the development of innovative non-invasive electrospun fibrous architectures with highly proper features capable of efficient gene therapy drug delivery in a spatial and temporal manner that can supplementarily provide physical support for cell development and guidance in accordance with the therapeutic purpose, its pharmacological properties and patient-specific therapeutic requirements. Moreover, further innovation in improving the physical and biological characteristics of electrospun-fibrous-architecture-mediated gene therapy drug delivery is pivotal in extending its use in precision genetic biomedicine, which can be translated beyond academic efforts into tangible personalized products that can substantially impact the gene therapy market and human quality of life.

## Figures and Tables

**Table 1:** Strengths, challenges and opportunities of viral and non-viral gene therapy drug delivery technologies [9,16,37,38,39].

	Viral Vectors	Non-Viral Vectors
*Types*	Retroviruses, lentiviruses, adenoviruses, adeno-associated viruses	Cationic polymers, cationic lipids, peptides, inorganic nanoparticles
*Strengths*	Compliant to both in vitro and in vivo tests; Advances position in clinical trials; Successful intracellular delivery; High transduction efficiency and strong promotion of gene expression	Compliant to both in vitro and in vivo tests; Loaded genetic material is protected from premature degradation during delivery; Viable for cell-specific and intracellular targeting; Low cost, simple manufacturing; High packaging abilities; Versatility in formulation
*Challenges*	May trigger immune responses (immunogenicity); Limited gene-packaging capacity; Expensive and laborious production, requiring extensive experience and particular safety measures; Difficulty of scaling up production	Ineffective and slow delivery, particularly in the case of endocytosis pathway; Intrinsic features of a carrier may perturb cell function or determine unpredictable toxic responses; Unfavorable disassembly and gene decomplexation kinetics; Limited in vivo transfection efficiency
*Opportunities*	Less-immunogenic vectors or circumvention of the immune system; Modulated tropism and specificity; Development of hybrid viral counterparts as well as new viral vectors; Advanced production methods	Advanced carriers capable of effectively co-delivering various cargoes; Modulation of target cell biology to improve delivery efficiency; Stimuli-responsive carriers; Employing direct diffusion to avoid endocytosis; Application of biomimetic functionalities inspired by viruses and exosomes

**Table 2:** PEI-based non-viral vectors under clinical trials.

Delivery System	Gene Therapy	Company/Institute	Targeted Application	Phase	Clinical Trial
***PEI***	BC-819/PEI	Anchiano Therapeutics, Israel, Ltd.	Superficial Bladder Cancer	II completed	NCT00595088
	DTA-H19	Anchiano Therapeutics, Israel, Ltd.	Pancreatic Neoplasms	II completed	NCT00711997
	CYL-02/Gemcitabine	University Hospital, Toulouse	Pancreatic Adenocarcinoma	II active	NCT02806687
***PEG-PEI-Cholesterol***	EGEN-001	Gynecologic Oncology Group	Ovarian, fallopian tubal and peritoneal cancers	II completed	NCT01118052
***PEIm***	DermaVir	Genetic Immunity	HIV Infection	II completed	NCT00711230

https://clinicaltrials.gov/ (accessed on 11 May 2022).

**Table 3:** Summary of the main advantages and drawbacks of strategies for formulating fibrous architectures for controlled gene therapy drug delivery [2,5,32,70,81,82].

Methods	Advantages	Drawbacks
***Encapsulation strategy***		
*Blend electrospinning*	Simple one-step method; Controlled and sustained release of genes	Risk of gene denaturation in some organic solvents; Uncontrollable gene distribution
*Coaxial * *electrospinning*	Optimal for core-shell/sheath structure; Feasible incorporation of genes within the nanofiber core and protection from organic solvents; Prolonged and controlled release of entrapped genes along with a lowered burst effect; Feasible for dual-loading/release cargoes	Complex spinneret construction; Improper interfacial interactions may generate defects in core-shell structure
*Emulsion electrospinning*	One-step method for formulating core-shell/sheath structure	Addition of surfactant to assure the optimal stability of emulsion
***Immobilization strategy***		
*Physical * *adsorption*	The easiest method for loading genes on the surface of fibers; The activity of loaded genes is not affected	Uncontrolled release profile
*Covalent * *immobilization*	Avoids unsafe solvents for genes; Improved surface properties of fibrous materials; Alternative method to generate multiple gene delivery	Use of external enzymes to obtain the controlled release profiles of genes; A crosslinker is necessary for prolonging gene release
*Plasma* *treatment*	Grafting of varied functionalities onto the nanofiber surface through using different gases; Immobilization of diverse genes on the treated surface; No requirement for solvents	Limited penetration depth into the nanofibers

**Table 4:** Representative electrospun-fibrous-architecture-mediated gene therapy drug delivery along with the targeted application and biological performances.

Electrospun Matrix	Non-Viral Vector	Therapeutic Gene	Targeted Application	Therapeutic Performances		Ref
				In Vitro	In Vivo	
*Natural polymer-based electrospun-fibrous-architecture-mediated gene therapy drug delivery*						
Alg	PP@KALA nanocomplexes	pVEGF	Skin wound healing	Prolonged gene release and long-term transgene expression of VEGF	Accelerated wound closure, promoted re-epithelization, reduced inflammatory response and enhanced neovascularization as well as skin wound healing in rats	[109]
Gel	pDNA/pAsp polyplexes	BMP-2	Tissue regeneration	Robust osteogenic ALP activity in C2C12 and MC3T3-E1 cells		[110]
*Synthetic polymer-based electrospun-fibrous-architecture-mediated gene therapy drug delivery*						
PLGA	siRNA/CS polyplexes	EGFP	Tissue engineering	Sustained siRNA delivery for 30 days; up to 50% silencing efficacy upon 48 h transfection with prolonged activity up to a 10-day duration		[83]
PCL	pDNA	Cdk2i, EGFPi	Cancer therapy, breast cancer	Sustained release of pDNA over 21 days; ∼40% decrease in the proliferation of the MCF-7 breast cancer cell line		[86]
PCLEEP	siRNA/CPP polyplexes	Col1A1 silencing	Regenerative medicine	Sustained release of siRNA over 28 days; 2–3-fold prolonged gene silencing of collagen type I production in HDFs	Significant reduction in fibrous capsule thickness 4 weeks after subcutaneous implantation in rats	[87]
PCL-PEG	MMP-LPEI/DNA complexes	pEGFP-N1	Local gene delivery for treating diabetic ulcers	Fast gene release in the presence of MMP-responsive peptides and good transfection and gene silencing effect	Significantly increased wound recovery in mice with diabetic ulcers	[98,99]
PCL/ PCLEEP	siRNA/MSN-PEI	COL1A1	Long-term non-viral gene silencing applications	Sustained release of siRNA extended up to 5 months	~45.8% reduction in fibrous capsule after 4 weeks of implantation in mice	[111]
PLLA/POSS NPs	pDNA	pAng-1	Angiogenic therapy	Sustained delivery of pAng over 35 days along with high transfection efficiency	Efficient promotion of angiogenesis and dermal wound healing rate	[112]
PLGA/HAp	Naked DNA; DNA/CS NPs	BMP-2 plasmid	Bone tissue regeneration	Protection of BMP-2 plasmids and enhanced cell attachment with negligible cytotoxicity	Maintained bioactivity of BMP-2 plasmid over 4 weeks in nude mice	[113]
PELCL	TMC-g-PEG-VAPG/ complexes	miRNA-145	Blood vessel regeneration	Sustained release of miRNA-145 for least 56 days; great transfection performance for regulating SMC cells		[114]
PVA	CHAT/pDNA NPs	pEGFP-N1	Localized gene therapy	Preserved DNA integrity and significant NCTC-929 cell uptake and gene expression		[115]
PVA	CHAT/pDNA NPs	pmiR-31	Wound regeneration	Enhanced endothelial and keratinocyte cell migration and improved angiogenic potential for both HaCaT and HMEC-1 cells	Significantly increased keratinocytes functionality as well as improved angiogenesis in mice	[116]
PET	PEI/siRNA complexes	TSP-2		Targeted and significant gene silencing in infiltrating AoSMCs		[117]
*Natural/synthetic polymer-based electrospun-fibrous-architecture-mediated gene therapy drug delivery*						
Alg/PCL	PEI/DNA polyplexes	pEGFP-C3	Tissue regeneration	Sustained release of polyplexes; increased and modulated in situ transfection activity; superior biocompatibility		[95,96,97]
PLLA/ Collagen I	pDNA/lipid complexes	pCMVβ and rhBMP-2	Bone tissue engineering	Robust (13-fold improvement) expression of rhBMP-2 mRNA following transfection in MC3T3 cells	Effective gene delivery and ability to simulate ectopic bone formation in mouse muscle pouches	[118]
PELCL, PCL/Gel	TMC-g-PEG-REDV polyplexes	miRNA-126	Blood vessel regeneration	Sustained release profile of miRNA-126 for 56 days; Significant improvement of VEC proliferation and down-regulation of SPRED-1 gene expression in VECs	Enhanced endothelialization after 8 weeks of replacing the carotid artery in rabbits	[119]
PCL-PIBMD/SF	SF MPs	pZNF580		Good cytocompatibility, along with the promotion of the proliferation, adhesion, spreading and migration of HUVECs	Diminished fibrous capsule formation and mitigated inflammatory reaction in rats	[120]

PVA: polyvinyl alcohol; PET: poly(ethylene terephthalate); PLGA/PEI-pDNA@KALA: cell-penetrating peptide; pVEGF: plasmid of vascular endothelial growth factor; pAsp: poly(aspartic acid); BMP-2: bone morphogenetic protein-2; COL1A1: collagen type I; CPP: cell penetrating peptide; pEGFP: plasmid of enhanced green fluorescent protein; TMC-g-PEG-VAPG: Val-Gly-Val-Ala-Pro-Gly-Cys, sequence peptide-modified trimethyl chitosan-g-poly(ethylene glycol); pAng-1: angiopoietin-1; pCMVβ: plasmid of cytomegalovirus encoding β-galactosidase; MSN-PEI: PEI-modified mesoporous silica nanoparticles; CHAT: 15 amino acid sequence peptide (NCHHHRRRWRRRHHHC-C); MMP: metalloproteinase; TSP-2: Thrombospondin-2; pZNF580: plasmid zinc finger protein 580.

## Data Availability

Not applicable.
